# One-Carbon Metabolism Pathway Gene Variants and Risk of Clear Cell Renal Cell Carcinoma in a Chinese Population

**DOI:** 10.1371/journal.pone.0081129

**Published:** 2013-11-21

**Authors:** Lei Zhang, Xiaoxin Meng, Xiaobing Ju, Hongzhou Cai, Pu Li, Qiang Cao, Pengfei Shao, Chao Qin, Changjun Yin

**Affiliations:** Department of Urology, the First Affiliated Hospital of Nanjing Medical University, Nanjing, China; Kunming Institute of Zoology, Chinese Academy of Sciences, China

## Abstract

**Background:**

One-carbon metabolism is the basement of nucleotide synthesis and the methylation of DNA linked to cancer risk. Variations in one-carbon metabolism genes are reported to affect the risk of many cancers, including renal cancer, but little knowledge about this mechanism is known in Chinese population.

**Methods:**

Each subject donated 5 mL venous blood after signing the agreement. The study was approved by the Institutional Review Board of the Nanjing Medical University, Nanjing, China. 18 SNPs in six one-carbon metabolism-related genes (*CBS, MTHFR, MTR, MTRR, SHMT1*, and *TYMS*) were genotyped in 859 clear cell renal cell carcinoma (ccRCC) patients and 1005 cancer-free controls by the Snapshot.

**Results:**

Strong associations with ccRCC risk were observed for rs706209 (*P* = 0.006) in *CBS* and rs9332 (*P* = 0.027) in *MTRR*. Compared with those carrying none variant allele, individuals carrying one or more variant alleles in these two genes had a statistically significantly decreased risk of ccRCC [*P* = 0.001, adjusted odds ratio (OR) = 0.73, 95% confidence interval (CI) = 0.06–0.90]. In addition, patients carrying one or more variant alleles were more likely to develop localized stage disease (*P* = 0.002, adjusted OR = 1.37, 95%CI = 1.11–1.69) and well-differentiated ccRCC (*P*<0.001, adjusted OR = 1.42, 95%CI = 0.87–1.68). In the subgroup analysis, individuals carrying none variant allele in older group (*P* = 0.007, adjusted OR = 0.67, 95%CI = 0.49–0.91), male group (*P* = 0.007, adjusted OR = 0.71, 95%CI = 0.55–0.92), never smoking group (*P* = 0.002, adjusted OR = 0.68, 95%CI = 0.53–0.88) and never drinking group (*P*<0.001, adjusted OR = 0.68, 95%CI = 0.53–0.88) had an increased ccRCC risk.

**Conclusions:**

Our results suggest that the polymorphisms of the one-carbon metabolism-related genes are associated with ccRCC risk in Chinese population. Future population-based prospective studies are required to confirm the results.

## Introduction

Renal cell carcinoma (RCC), one lethal urologic cancer accounting for 2% of all cancer deaths, is the third most common cancer of the genitourinary tract, just next to prostate and bladder cancer [1]. The most common subtype of RCC is the clear cell type (ccRCC), accounting for 75% of all cases. The incidence of RCC has increased rapidly worldwide over the past few decades [2]. Although the exact causes of RCC remain unknown, a few well-established lifestyle risk factors have been identified, including: cigarette smoking, obesity, hypertension and diabetes [3]. Recent studies have demonstrated that genetic polymorphisms of candidate genes were associated with susceptibility and/or prognosis of cancers, including RCC [4], [5], [6], [7].

One-carbon metabolism refers to a system of interdependent metabolic pathways facilitating the transfer of one-carbon units, which are the basement of nucleotide synthesis and the methylation of DNA. Methylation is responsible for gene expression control, chromatin structure stability and the maintenance of genomic stability. It has been proved that tumor-suppressor genes are silenced by hypermethylation of the promoter region in various cancers, leading to carcinogenesis and cancer progression [8]. In RCC, genes encoding von Hippel-Lindan (VHL), E-cadherin and RASSF1A have been reported to be inactivated by promoter hypermethylation [9], [10], [11]. DNA hypomethylation begets chromosomal instability as a result of changes in chromatin structure [12], [13].

The connection between the polymorphisms in one-carbon metabolism pathway genes, such as methylenetetrahydrofolate reductase (*MTHFR*), has been widely studied in various cancers [14], [15], [16], [17], [18]. The *MTHFR* promoter polymorphism rs3737965 has been intensively studied, but the results were conflicting, especially in lung cancer [16], [19], [20], [21], [22]. Besides, two large case-control studies for RCC have been conducted in Europe to analyze the candidate genes in one-carbon metabolism pathway. The studies found the association between the one-carbon metabolism pathway genes polymorphisms and RCC risk [14], [15]. However, there are some discrepancies in these two studies, as evidenced by the confusing connection between the *TYMS* polymorphisms and RCC risk. Given the importance of this pathway in carcinogenesis, ethnic differences and the discrepancies in previous studies, the present case-control study was conducted to define the association between the one-carbon metabolism pathway genes polymorphisms and the ccRCC risk in Chinese population, through testing the polymorphism in six key genes (*CBS, MTHFR, MTR, MTRR, SHMT1*, and *TYMS*) in this pathway.

## Materials and Methods

### Study Subjects

859 ccRCC patients and 1005 cancer-free controls were enrolled in our case-control study. All subjects were genetically unrelated ethnic Han Chinese recruited from May 2004 to January 2012 at the First Affiliated Hospital of Nanjing Medical University, Nanjing, China, the Affiliated Hospital of Medical College Qingdao University, Qingdao, China and the Third Affiliated Hospital of Soochow University, Changzhou, China [23]. The cases were newly diagnosed with incident ccRCC and had been histopathologically confirmed without history of other malignancies and previous chemotherapy or radiotherapy. A standard questionnaire was administered through face-to-face interviews by trained interviewers to collect demographic data and related information. Each subject donated 5 mL venous blood after signing the agreement. The disease was classified by the World Health Organization criteria and stage according to the American Joint Committee on Cancer (AJCC) TNM (tumor-node-metastasis) classification. The Fuhrman scale was used to assess tumor nuclear grade and the disease stage was divided into two subgroups (localized group: stage I and stage II and advanced group: stage III and stage IV). The controls were individuals without history of cancer who were seeking health care in the outpatient departments matched to the cases' sex and age (±5 years) on frequency. We subdivided the patients and controls into two groups (younger group: age ≤57 and older group: age >57) according to the mean age of the cohort (57 years old). Smokers were defined as those who smoked daily for more than 1 year. Drinkers were those who drank at least 3 times per week and more than 6 months. The study was approved by the Institutional Review Board of the Nanjing Medical University, Nanjing, China.

### DNA extraction and genotyping

Genomic DNA was seperated and purified from leucocytes of peripheral blood by proteinase K digestion and phenol/chloroform extraction. Genotyping of the polymorphism was performed by the Snapshot. The SNaPshot SNP assay was performed to detect the dimorphism at the two SNP loci. PCR was performed in a 20 µl reaction mixture containing 1 µl (10 ng) of template DNA, 1 µM of each primer, 0.3 mM of each deoxynucleotide triphosphate, 3.0 mM of MgCl2, and 1 U HotStarTaq polymerase (Qiagen Inc., USA) with 1× HotStarTaq buffer. The PCR program consisted of an initial melting step of 15 minutes at 95°C; 11 cycles of 20 seconds at 94°C, 40 seconds at 65°C-0.5°C/cycle, and 90 seconds at 72°C; 24 cycles of 20 seconds at 94°C, 30 seconds at 59°C, and 90 seconds at 72°C; and a final elongation step of 2 minutes at 72°C. To purify the PCR products, 1 U SAP and 1 U Exonuclease I were mixed with 10 µl PCR product for 1 hour at 37°C and 15 minutes at 75°C.The extension reaction was performed in a 10 µl reaction mixture containing 5 µl of the SNaPshot Multiplex Kit (Applied Biosystems, USA), 2 µl of purified PCR products, 0.8 µM of the extension reaction primer, and 2 µl water. The PCR program was 1 minute at 96°C; 28 cycles of 10 seconds at 96°C, 5 seconds at 50°C, and 30 seconds at 60°C; and 4°C as the holding temperature. Finally, 10 µl of the extension product was purified with 1 U SAP for 1 hour at 37°C and inactivated for 15 minutes at 75°C.The resulting data were analyzed with an ABI3130XL sequencer and GeneMapper™ 4.0 Software (Applied Biosystems, Co. Ltd., USA). All the sequences of primers for each SNP are listed in Table S1. Positive controls by sequencing and negative controls were used to ensure the accuracy of genotyping. To ensure quality control (QC), genotyping was performed by researchers blinded to the case/control status of the subjects, and a random sample of 5% of the cases and controls was genotyped twice by different persons, with a reproducibility of 100%.

### Statistical analysis

Statistical analysis were performed by software SAS 9.1.3 (SAS Institute, Cary, NC). Allele frequencies were tested against departure from Hardy-Weinberg equilibrium through goodness-of-fit χ2 test before analysis. Differences in the distributions of demographic characteristics, selected variables, and frequencies of genotypes between cases and controls were evaluated by Student's t-test (for continuous variables) or χ2-test (for categorical variables). OR and 95% CIs from unconditional logistic regression analysis with the adjustment for possible confounders were used to estimate the association between the polymorphism and the risk of ccRCC. All of the statistical tests were two-sided and *P*<0.05 was considered statistically significant.

## Results

### Characteristics of ccRCC patients and controls

Clinical and pathologic characteristics of the 859 cases and 1005 controls are presented in Table 1 (negative results listed in Table S2). No significant differences were found between the cases and controls in regards to age, gender and drinking status (all *P*>0.05). However, there were more high BMI, smoking habit, hypertension and diabetes subjects in ccRCC patients than those in controls (*P* = 0.003, *P* = 0.040, *P*<0.001 and *P* = 0.040, respectively). Among 859 ccRCC cases, 577 (67.2%) patients were diagnosed with stage I disease, 170 (19.8%) patients with stage II, 57 (6.7%) patients with stage III, and 55 (6.4%) patients with stage IV. The percent of nuclear grade from I to IV was 20.5%, 54.7%, 19.9%, and 4.9%, respectively.

**Table 1:** Distribution of selected variables between the clear cell renal cell carcinoma cases and control subjects.

Variables	Cases (n = 859)		Controls (n = 1005)		*P*-value^a^
	N	%	N	%	
Age (years) (mean ± SD)	57.0±11.7		57.2±12.4		0.758
BMI (kg/m2) (mean ± SD)	24.2±2.9		23.8±3.2		**0.003**
Gender					
Male	557	64.8	688	68.5	0.098
Female	302	35.2	317	31.5	
Smoking status					
Never	530	61.7	666	66.3	**0.040**
Ever	329	38.3	339	33.7	
Drinking status					
Never	619	72.1	757	75.3	0.110
Ever	240	27.9	248	24.7	
Hypertension					
No	522	60.8	675	67.2	**<0.001**
Yes	337	39.2	251	25.0	
Diabetes					
No	748	87.6	870	86.6	**<0.001**
Yes	111	12.4	56	5.6	
Clinical stage					
I	577	67.2			
II	170	19.8			
III	57	6.7			
IV	55	6.4			
Grade					
I	176	20.5			
II	470	54.7			
III	171	19.9			
IV	42	4.9			

a Student's t-test for age and BMI distributions between cases and controls; two-sided χ2-test for others selected variables between cases and controls.

### Association of SNPs with the risk of ccRCC

As shown in Table 2, except the rs1788484 in *CBS* gene and rs10520873 in *MTRR* gene, the genotype distributions of all other SNPs were in agreement with Hardy-Weinberg equilibrium in controls. Therefore, the rs1788484 and rs10520873 were excluded for further analysis. Table 2 shows ccRCC risks were associated with each single polymorphism. We presented each of the associations in additive, dominative and recessive models. In *CBS* gene, the genotype of rs706209 polymorphism distribution between cases and controls was significantly different (*P* = 0.006). In the dominant model, when compared with the homozygous wild-type reference group, TC+CC genotype was significantly associated with a decreased risk of ccRCC (*P* = 0.002, adjusted OR = 0.76, 95%CI = 0.62–0.91). In *MTRR* gene, the genotype of rs9332 polymorphism distribution between cases and controls was significantly different (*P* = 0.027). In the dominant model, CT+TT genotype was significantly associated with a decreased risk of ccRCC (*P* = 0.010, adjusted OR = 0.74, 95%CI = 0.60–0.92), when compared with the CC group. The rs2966952 seemed to be a bordering positive result. In the recessive models, TT genotype was slightly associated with an increased risk of ccRCC (*P* = 0.050, adjusted OR = 1.34, 95%CI = 1.00–1.80), compared with the CC+CT genotype. In *TYMS* gene, the genotype of rs2853741 polymorphism distribution between cases and controls had no significantly difference (*P* = 0.096). In the dominant model, TC+CC genotype was significantly associated with an increased risk of ccRCC (*P* = 0.032, adjusted OR = 1.25, 95%CI = 1.02–1.53), when compared with the TT group. Similarly, for the rs699517, the polymorphism distribution between cases and controls had no significantly difference (*P* = 0.108). In the recessive models, CC genotype was seemed to be associated with an increased risk of ccRCC (*P* = 0.172, adjusted OR = 1.25, 95%CI = 1.03–1.51).

**Table 2:** Polymorphism in one-carbon metabolism genes and clear cell renal cell carcinoma risk.

	Position	MAF	Cases, n (%)	Controls, n (%)	*P*-value^a^	Adjusted OR (95% CI)^b^	*P* trend^a^	HWE
*CBS*								
rs1788484	promoter	0.222						
CC			535(62.3)	543(54.0)	**<0.001**	Ref.	**0.022**	**<0.001**
CT			275(32.0)	425(42.3)		0.62(0.51–0.76)		
TT			49(5.7)	37(3.7)		1.27(0.79–2.20)		
TT vs. CC+CT					**0.038**	1.53(0.97–2.41)		
CT+TT vs. CC					**<0.001**	**0.67(0.55–0.82)**		
rs706209	3′UTR	0.329						
TT			401(46.7)	396(39.4)	**0.006**	Ref.	**0.007**	0.379
TC			358(41.7)	480(47.8)		**0.76(0.62–0.92)**		
CC			100(11.6)	129(12.8)		0.74(0.55–1.01)		
CC vs. TT+TC					0.434	0.87(0.65–1.16)		
TC+CC vs. TT					**0.002**	**0.76(0.62–0.91)**		
*MTRR*								
rs2966952	promoter	0.333						
CC			371(43.2)	463(46.1)	0.120	Ref.	0.064	0.978
CT			374(43.5)	438(43.6)		1.07(0.88–1.31)		
TT			114(13.3)	104(10.4)		**1.40(1.03–1.91)**		
TT vs. CC+CT					**0.050**	**1.34(1.00–1.80)**		
CT+TT vs. CC					0.213	1.13(0.93–1.36)		
rs9332	3′UTR	0.188						
CC			651(75.8)	708(70.5)	**0.027**	Ref.	**0.007**	0.434
CT			196(22.8)	275(27.4)		**0.75(0.60–0.93)**		
TT			12(1.4)	22(2.2)		0.65(0.31–1.33)		
TT vs. CC+CT					0.203	0.70(0.34–1.43)		
CT+TT vs. CC					**0.010**	**0.74(0.60–0.92)**		
rs10520873	3′UTR	0.311						
AA			593(69.0)	648(64.5)	0.063	Ref.	0.098	**0.032**
AG			240(27.9)	331(32.9)		**0.79(0.64–0.97)**		
GG			26(3.0)	26(2.6)		1.15(0.65–2.04)		
GG vs. AA+AG					0.566	1.24(0.70–2.19)		
AG+GG vs. AA					**0.038**	**0.82(0.67–1.00)**		
*TYMS*								
rs2853741	promoter	0.411						
TT			264(30.7)	356(35.4)	0.096	Ref.	**0.046**	0.982
TC			439(51.1)	484(48.2)		**1.25(1.01–1.54)**		
CC			156(18.2)	165(16.4)		1.24(0.93–1.64)		
CC vs. TT+TC					0.321	1.08(0.84–1.39)		
TC+CC vs. TT					**0.032**	**1.25(1.02–1.53)**		
rs699517	3′UTR	0.3						
TT			420(48.9)	537(53.4)	0.108	Ref.		
TC			358(41.7)	391(38.9)		**1.25(1.02–1.52)**	**0.035**	0.619
CC			81(9.4)	77(7.7)		1.26(0.89–1.78)		
CC vs. TT+TC					0.172	**1.25(1.03–1.51)**		
TC+CC vs. TT					0.051	1.15(0.82–1.61)		

a Two-sided χ2-test for either genotype distributions or trend between the cases and controls.

b genotype-specific ORs were adjusted for age, gender, BMI, smoking status, drinking status, diabetes and hypertension in logistic regression model.

### Combined Analysis of Polymorphisms

Rs706209 or rs9332 alone being associated with ccRCC risk, we combined these two polymorphisms based on the number of the variant alleles. As shown in Table 3, statistically significant difference was obviously found between the subgroups and the risk of ccRCC. Meanwhile, individuals carrying 1, 2, 3, or 4 variant alleles in these two genes were associated with a statistically significantly decreased risk of ccRCC compared with individuals carrying none variant alleles (*P* = 0.001, adjusted OR = 0.73, 95%CI = 0.06–0.90).

**Table 3:** Combined genotype frequencies among the case patients and control subjects and their collection to clear cell renal cell carcinoma.

Variables	Case (n = 859)		Controls (n = 1005)		*P*-value^a^	Adjusted OR (95% CI)^b^
	n	%	n	%		
rs706209 and rs9332						
Number of risk allele						
0	304	35.4	286	28.5		
1	358	41.7	432	43.0	**0.022**	**0.79(0.64–0.99)**
2	171	19.9	237	23.6	**0.003**	**0.68(0.52–0.88)**
3	26	3.0	49	4.9	**0.006**	**0.48(0.29–0.81)**
4	0	0	1	0		
Recombined groups						
0	304	35.4	286	28.5		
1–4	555	64.6	719	71.5	**0.001**	**0.73(0.06–0.90)**

a Two-sided χ2 test for the distributions of genotypes.

b Adjusted for age, gender, BMI, smoking status, drinking status, hypertension and diabetes in logistic regression model.

### Stratified Analysis of the Two Polymorphisms and Clinicopathologic Characteristics and Risk of ccRCC

We further evaluated the association between the combined genotypes of rs706209 and rs9332 polymorphisms and clinicopathologic characteristics of ccRCC. These two polymorphisms were combined based on the number of the variant alleles. As shown in Table 4, a significantly increased risk appeared in ccRCC patients with localized stage (*P* = 0.002, adjusted OR = 1.37, 95%CI = 1.11–1.69) and in patients with well-differentiated ccRCC (*P*<0.001, adjusted OR = 1.42, 95%CI = 1.14–1.76).

**Table 4:** Association between the combined genotypes of rs706209 and rs9332 polymorphisms and clinicopathologic characteristics of clear cell renal cell carcinoma.

Variables	Risk allele				*P*-value^a^	Adjusted OR (95% CI)^b^
	0		1–4			
	n	%	n	%		
Control (n = 1005)	286(28.5)		719(71.5)			Ref.
Case (n = 859)						
Clinical stage						
Localized (I+II)	265(35.5)		482 (64.5)		**0.002**	**1.37 (1.11–1.69)**
Advanced (III+IV)	39 (34.8)		73 (65.2)		0.160	1.30 (0.86–1.98)
Grade						
Well- differentiated (I+II)	234 (36.2)		412 (63.8)		**<0.001**	**1.42 (1.14–1.76)**
Moderately and Poorly differentiated(III+IV)	70 (32.9)		143 (67.1)		0.199	1.21 (0.87–1.68)

a Two-sided χ2 test for the distributions of genotypes.

b Adjusted for age, gender, BMI, smoking status, drinking status, hypertension and diabetes in logistic regression model.

As shown in Table 5, the analysis was stratified by age, gender, smoking status, and drinking status. Individuals carrying none variant allele in older group (*P* = 0.007, adjusted OR = 0.67, 95%CI = 0.49–0.91), male group (*P* = 0.007, adjusted OR = 0.71, 95%CI = 0.55–0.92), never smoking group (*P* = 0.002, adjusted OR = 0.68, 95%CI = 0.53–0.88) and never drinking group (*P*<0.001, adjusted OR = 0.68, 95%CI = 0.53–0.88) had an increased ccRCC risk.

**Table 5:** Association between the combined genotypes of rs706209 and rs9332 polymorphisms and clear cell renal cell carcinoma in stratified analysis.

Variables	Risk allele				*P*-value^a^	Adjusted OR (95% CI)^b^
	0		1–4			
	Case(n,%)	control(n,%)	case(n,%)	control(n,%)		
Age						
< = 57	161(36.2)	164(30.4)	284(63.8)	376(69.6)	0.054	0.78(0.60–1.02)
>57	143(34.5)	122(26.2)	271(65.5)	343(73.8)	**0.007**	**0.67(0.49–0.91)**
Gender						
Male	192(34.5)	188(27.3)	365(65.5)	500(72.7)	**0.007**	**0.71(0.55–0.92)**
Female	121(37.1)	98(30.9)	190(62.9)	219(69.1)	0.105	0.77(0.55–1.08)
Smoke						
Never	193(36.4)	187(28.1)	337(63.6)	479(71.9)	**0.002**	**0.68(0.53–0.88)**
Ever	111(33.7)	99(29.2)	218(66.3)	240(70.8)	0.207	0.83(0.59–1.17)
Drink						
Never	229(37.0)	202(26.7)	390(63.0)	555(73.3)	**<0.001**	**0.68(0.53–0.88)**
Ever	75(31.3)	84(33.9)	165(68.8)	164(66.1)	0.537	0.83(0.59–1.17)

a Two-sided χ2 test for the distributions of genotypes.

b Adjusted for age, gender, BMI, smoking status, drinking status, hypertension and diabetes in logistic regression model.

## Discussion

Our study supports a role that *CBS*, *MTRR*, and *TYMS* play in modifying ccRCC risk. Meanwhile, we also notice the discrepancies between our findings and the results reported by the two European studies. Moore *et al.* reported that *MTHFR* and *TYMS* had effect on RCC risk, while *CBS*, *MTR*, or *MTRR* had no [14]. Gibson *et al.* reported that the strongest association between RCC risk and *MTHFR*, not *CBS*, *MTR*, *MTRR*, *SHMT1* or *TYMS*, was observed [15]. The discrepancies might be due to the different aspect of study, different ethnic population and other unknown factors. Our current study is focus on the ccRCC, which is one part of RCC.The pathologic characteristics of ccRCC in our study may be relatively simple, when compared with these two European articals. In the dbSNP database, the minor allele frequency (MAF) of rs706209 was T allele  = 0.403 in European, but the MAF in Chinese was C allele  = 0.402, close to our data C allele  = 0.482. As involved in one-carbon metabolism pathway, the *CBS* gene encodes cystathionine beta synthase (CBS), which is the central enzyme in the transsulfuration pathway irreversibly metabolizing homocysteine (Hcy) to cystathionine [24]. Evidences showed that functional *CBS* gene SNP could impair *CBS* gene function, leading to an increase in the concentration on tHcy that further influenced aberrant DNA methylation patterns and *CBS* gene SNP had associations with lung cancer, colorectal cancer and head and neck squamous cell carcinoma [16], [25], [26]. Whether rs706209 has the similar influence on the *CBS* gene function needs further functional studies. MTRR catalyzes reductive methylation of cob (II)alamin by using SAM as a methyl donor to reactivate MTR. Thus, MTRR may act as a key regulator of the homocysteine conversion to methionine [27]. Our study demonstrated that CT+TT genotype of rs9332 was significantly associated to a decreased risk of ccRCC. Other report demonstrated that rs9332 in *MTRR* had connections with spina bifida and conotruncal heart defects [28]. Although little information about the function of this polymorphism was known, it is known that 3′UTR of the *MTRR* gene may influence miRNA or siRNA binding target site, causing the degradation of the mRNA, or inhibiting translation initiation. This polymorphism has a protective role in the risk of ccRCC. More researches should be done to reveal the relationship between them.

Moreover, we found that *TYMS* SNPs (rs2966952 and rs699517) had some of connections with ccRCC risk. Skibola *et al.* reported that C allele in rs699517 was approximately 100% correlated with *TYMS* 1496 insertion and T allele in rs699517 was also approximately 100% correlated with *TYMS* 1496 deletion. The *TYMS* 1496 deletion polymorphism was associated with decreased mRNA stability and low expression in tumor tissue than the wild type polymorphism [29], [30], [31]. Evidence demonstrated that the level of thymidylate synthetase (TS) activity was correlated with both the progression of the stage and the increase of the grade of RCC, and the activity of TS was approximately 5-fold higher in RCC compared with normal kidney [32].

There were several limitations in our present study. Firstly, our standard questionnaire contained no environmental factors, such as occupational exposure, and personal habits, such as diet and physical activity. So our study may have a limited statistical power, such as that we could not do further analysis in gene-environment interaction. Secondly, more and more studies indicated that gene-gene interaction may also have contribution to the risk of cancer [33], [34]. In the current study, we did little analysis about the interaction among genes in the one-carbon metabolism pathway. So the results may exist some interaction bias. Furthermore, our study was designed as a hospital-based study, so the possibility of selection bias of subjects could not be ruled out. Thirdly, the genotype distributions of rs1788484 in *CBS* gene and rs10520873 in *MTRR* gene were departed from Hardy-Weinberg equilibrium in controls. The probability of genotyping error was very low. Meanwhile, Galbiatti *et al.* also reported that polymorphism of *CBS* gene was not in Hardy-Weinberg equilibrium [26]. The departure from the Hardy-Weinberg equilibrium may result from selection bias, disease model adopted, and evolutionary factors which may influence changes in the genotype frequencies [35], [36]. On the other hand, this disequilibrium should be expected, in the case that it reflected biological and genetic characteristics in complex disease models [37].

In summary, the data indicates that the common variation in *CBS*, *MTRR* and *TYMS* may significantly modify ccRCC risk.

## Supporting Information

Table S1 All the sequences of primers for each SNP.(DOC).
(DOC)

Table S2 Polymorphism in one-carbon metabolism genes and clear cell renal cell carcinoma. (DOC).
(DOC)

## References

[1] Siegel R, Ward E, Brawley O, Jemal A (2011) Cancer statistics, 2011: the impact of eliminating socioeconomic and racial disparities on premature cancer deaths. CA Cancer J Clin 61: 212–236. 21685461 10.3322/caac.20121

[2] Chow WH, Dong LM, Devesa SS (2010) Epidemiology and risk factors for kidney cancer. Nat Rev Urol 7: 245–257. 20448658 10.1038/nrurol.2010.46 PMC3012455

[3] Mathew A, Devesa SS, Fraumeni JF Jr, Chow WH (2002) Global increases in kidney cancer incidence, 1973–1992. Eur J Cancer Prev 11: 171–178. 11984136 10.1097/00008469-200204000-00010

[4] Hirata H, Hinoda Y, Nakajima K, Kikuno N, Suehiro Y, et al (2009) The bcl2 -938CC genotype has poor prognosis and lower survival in renal cancer. J Urol 182: 721–727. 19539330 10.1016/j.juro.2009.03.081

[5] Lin J, Horikawa Y, Tamboli P, Clague J, Wood CG, et al (2010) Genetic variations in microRNA-related genes are associated with survival and recurrence in patients with renal cell carcinoma. Carcinogenesis 31: 1805–1812. 20732906 10.1093/carcin/bgq168 PMC2981457

[6] Kleinrath T, Gassner C, Lackner P, Thurnher M, Ramoner R (2007) Interleukin-4 promoter polymorphisms: a genetic prognostic factor for survival in metastatic renal cell carcinoma. J Clin Oncol 25: 845–851. 17327605 10.1200/JCO.2006.07.8154

[7] Kawai Y, Sakano S, Korenaga Y, Eguchi S, Naito K (2007) Associations of single nucleotide polymorphisms in the vascular endothelial growth factor gene with the characteristics and prognosis of renal cell carcinomas. Eur Urol 52: 1147–1155. 17287073 10.1016/j.eururo.2007.01.073

[8] Teodoridis JM, Strathdee G, Plumb JA, Brown R (2004) CpG-island methylation and epigenetic control of resistance to chemotherapy. Biochem Soc Trans 32: 916–917. 15506923 10.1042/BST0320916

[9] Shinojima T, Oya M, Takayanagi A, Mizuno R, Shimizu N, et al (2007) Renal cancer cells lacking hypoxia inducible factor (HIF)-1alpha expression maintain vascular endothelial growth factor expression through HIF-2alpha. Carcinogenesis 28: 529–536. 16920734 10.1093/carcin/bgl143

[10] Kawakami T, Okamoto K, Ogawa O, Okada Y (2003) Multipoint methylation and expression analysis of tumor suppressor genes in human renal cancer cells. Urology 61: 226–230. 12559313 10.1016/s0090-4295(02)02110-6

[11] Morrissey C, Martinez A, Zatyka M, Agathanggelou A, Honorio S, et al (2001) Epigenetic inactivation of the RASSF1A 3p21.3 tumor suppressor gene in both clear cell and papillary renal cell carcinoma. Cancer Res 61: 7277–7281. 11585766

[12] Eden A, Gaudet F, Waghmare A, Jaenisch R (2003) Chromosomal instability and tumors promoted by DNA hypomethylation. Science 300: 455. 12702868 10.1126/science.1083557

[13] Feinberg AP (2007) Phenotypic plasticity and the epigenetics of human disease. Nature 447: 433–440. 17522677 10.1038/nature05919

[14] Moore LE, Hung R, Karami S, Boffetta P, Berndt S, et al (2008) Folate metabolism genes, vegetable intake and renal cancer risk in central Europe. Int J Cancer 122: 1710–1715. 18098291 10.1002/ijc.23318

[15] Gibson TM, Brennan P, Han S, Karami S, Zaridze D, et al (2011) Comprehensive evaluation of one-carbon metabolism pathway gene variants and renal cell cancer risk. PLoS One 6: e26165. 22039442 10.1371/journal.pone.0026165 PMC3198392

[16] Flores KG, Stidley CA, Mackey AJ, Picchi MA, Stabler SP, et al (2012) Sex-specific association of sequence variants in CBS and MTRR with risk for promoter hypermethylation in the lung epithelium of smokers. Carcinogenesis 33: 1542–1547. 22665368 10.1093/carcin/bgs194 PMC3499054

[17] Shen M, Rothman N, Berndt SI, He X, Yeager M, et al (2005) Polymorphisms in folate metabolic genes and lung cancer risk in Xuan Wei, China. Lung Cancer 49: 299–309. 15922487 10.1016/j.lungcan.2005.04.002

[18] Curtin K, Slattery ML, Ulrich CM, Bigler J, Levin TR, et al (2007) Genetic polymorphisms in one-carbon metabolism: associations with CpG island methylator phenotype (CIMP) in colon cancer and the modifying effects of diet. Carcinogenesis 28: 1672–1679. 17449906 10.1093/carcin/bgm089 PMC2442467

[19] Cui LH, Yu Z, Zhang TT, Shin MH, Kim HN, et al (2011) Influence of polymorphisms in MTHFR 677 C–>T, TYMS 3R–>2R and MTR 2756 A–>G on NSCLC risk and response to platinum-based chemotherapy in advanced NSCLC. Pharmacogenomics 12: 797–808. 21605004 10.2217/pgs.11.27

[20] Shi Q, Zhang Z, Li G, Pillow PC, Hernandez LM, et al (2005) Sex differences in risk of lung cancer associated with methylene-tetrahydrofolate reductase polymorphisms. Cancer Epidemiol Biomarkers Prev 14: 1477–1484. 15941959 10.1158/1055-9965.EPI-04-0905

[21] Boccia S, Boffetta P, Brennan P, Ricciardi G, Gianfagna F, et al (2009) Meta-analyses of the methylenetetrahydrofolate reductase C677T and A1298C polymorphisms and risk of head and neck and lung cancer. Cancer Lett 273: 55–61. 18789576 10.1016/j.canlet.2008.07.026

[22] Mao R, Fan Y, Jin Y, Bai J, Fu S (2008) Methylenetetrahydrofolate reductase gene polymorphisms and lung cancer: a meta-analysis. J Hum Genet 53: 340–348. 18340404 10.1007/s10038-008-0262-6

[23] Qin C, Cao Q, Ju X, Wang M, Meng X, et al (2012) The polymorphisms in the VHL and HIF1A genes are associated with the prognosis but not the development of renal cell carcinoma. Ann Oncol 23: 981–989. 21778301 10.1093/annonc/mdr325

[24] Yi P, Melnyk S, Pogribna M, Pogribny IP, Hine RJ, et al (2000) Increase in plasma homocysteine associated with parallel increases in plasma S-adenosylhomocysteine and lymphocyte DNA hypomethylation. J Biol Chem 275: 29318–29323. 10884384 10.1074/jbc.M002725200

[25] Le Marchand L, Donlon T, Hankin JH, Kolonel LN, Wilkens LR, et al (2002) B-vitamin intake, metabolic genes, and colorectal cancer risk (United States). Cancer Causes Control 13: 239–248. 12020105 10.1023/a:1015057614870

[26] Galbiatti AL, Ruiz MT, Raposo LS, Maniglia JV, Pavarino-Bertelli EC, et al (2010) The association between CBS 844ins68 polymorphism and head and neck squamous cell carcinoma risk - a case-control analysis. Arch Med Sci 6: 772–779. 22419938 10.5114/aoms.2010.17094 PMC3298348

[27] Leclerc D, Wilson A, Dumas R, Gafuik C, Song D, et al (1998) Cloning and mapping of a cDNA for methionine synthase reductase, a flavoprotein defective in patients with homocystinuria. Proc Natl Acad Sci U S A 95: 3059–3064. 9501215 10.1073/pnas.95.6.3059 PMC19694

[28] Shaw GM, Lu W, Zhu H, Yang W, Briggs FB, et al (2009) 118 SNPs of folate-related genes and risks of spina bifida and conotruncal heart defects. BMC Med Genet 10: 49. 19493349 10.1186/1471-2350-10-49 PMC2700092

[29] Skibola CF, Forrest MS, Coppede F, Agana L, Hubbard A, et al (2004) Polymorphisms and haplotypes in folate-metabolizing genes and risk of non-Hodgkin lymphoma. Blood 104: 2155–2162. 15198953 10.1182/blood-2004-02-0557

[30] Mandola MV, Stoehlmacher J, Zhang W, Groshen S, Yu MC, et al (2004) A 6 bp polymorphism in the thymidylate synthase gene causes message instability and is associated with decreased intratumoral TS mRNA levels. Pharmacogenetics 14: 319–327. 15115918 10.1097/00008571-200405000-00007

[31] Ulrich CM, Curtin K, Potter JD, Bigler J, Caan B, et al (2005) Polymorphisms in the reduced folate carrier, thymidylate synthase, or methionine synthase and risk of colon cancer. Cancer Epidemiol Biomarkers Prev 14: 2509–2516. 16284371 10.1158/1055-9965.EPI-05-0261

[32] Mizutani Y, Wada H, Yoshida O, Fukushima M, Nonomura M, et al (2003) Significance of thymidylate synthase activity in renal cell carcinoma. Clin Cancer Res 9: 1453–1460. 12684419

[33] Zhong R, Liu L, Zou L, Sheng W, Zhu B, et al (2013) Genetic variations in the TGFbeta signaling pathway, smoking and risk of colorectal cancer in a Chinese population. Carcinogenesis 34: 936–942. 23275154 10.1093/carcin/bgs395

[34] Liu L, Wu C, Wang Y, Zhong R, Wang F, et al (2011) Association of candidate genetic variations with gastric cardia adenocarcinoma in Chinese population: a multiple interaction analysis. Carcinogenesis 32: 336–342. 21148629 10.1093/carcin/bgq264

[35] Xu J, Turner A, Little J, Bleecker ER, Meyers DA (2002) Positive results in association studies are associated with departure from Hardy-Weinberg equilibrium: hint for genotyping error? Hum Genet 111: 573–574. 12516594 10.1007/s00439-002-0819-y

[36] Llorca J, Prieto-Salceda D, Combarros O, Dierssen-Sotos T, Berciano J (2005) [Competing risks of death and Hardy-Weinberg equilibrium in case-control studies of gene-disease association]. Gac Sanit 19: 321–324. 16050969 10.1157/13078032

[37] Wittke-Thompson JK, Pluzhnikov A, Cox NJ (2005) Rational inferences about departures from Hardy-Weinberg equilibrium. Am J Hum Genet 76: 967–986. 15834813 10.1086/430507 PMC1196455

