# Supplementary material for: One-Carbon Metabolism Pathway Gene Variants and Risk of Clear Cell Renal Cell Carcinoma in a Chinese Population
Source: PLoS One. 2013 Nov 21;8(11):e81129. doi: 10.1371/journal.pone.0081129 (PMC3837692; doi:10.1371/journal.pone.0081129)
Supplement: Table S1 — All the sequences of primers for each SNP.(DOC). (DOC) [file pone.0081129.s001.doc]

**Table S1.** All the sequences of primers for each SNP.

| Gene name | SNP | 2nd-PCRP | 1st-PCRP | UEP_SEQ |
| --- | --- | --- | --- | --- |
| ***CBS*** | rs1788484 | ACGTTGGATGAGCTGCGCGGGCCGGAGGA | ACGTTGGATGTCTGGGCAGAGGAACGTGCT | AGGCCGGAGGAGGCTGCGGA |
|  | rs706209 | ACGTTGGATGGACGGGAGGGGAAATGATTG | ACGTTGGATGAAAGTTGTGTAGGCACCACC | GAGGGGAAATGATTGCTTCAC |
| ***MTHFR*** | rs3737965 | ACGTTGGATGACCCCCGGCAACGCCTCTCT | ACGTTGGATGAGGAGCTGGTAAGAAGCCG | CCCCCGGCAACGCCTCTCTCAGTCC |
|  | rs4846049 | ACGTTGGATGTGTTTTGCCTGTACTGCACG | ACGTTGGATGAACCAAGGCAGCCTCCAGA | ACGTACTGCACGGGCTCCAAG |
|  | rs4846048 | ACGTTGGATGCAGAAGCAGTTAGTTCTGAC | ACGTTGGATGGTTTGGTGGTGGCTTCTTCC | GCAGTTAGTTCTGACACCAACAA |
| ***MTR*** | rs1050993 | ACGTTGGATGTTCTTCGTCTTCATCCTTCG | ACGTTGGATGAGATGGTGGTGGCAATAGTC | AAACCAGGACTCCAATG |
|  | rs6676866 | ACGTTGGATGGGCTTTGCTAGGTTGAAGAC | ACGTTGGATGTCCTACCTTTCCCTCCAATC | TGTGGAAGACTTGTTGAC |
|  | rs2282368 | ACGTTGGATGACCCCTTATCTTAACCCAG | ACGTTGGATGTGGGTGACAGAGCCGTATG | ACCCAGATATTCCTTTCTATT |
| ***MTRR*** | rs2966952 | ACGTTGGATGGAAGGTCATTTCCATTTTTCG | ACGTTGGATGACGACAACCTGAGCCTTTC | AATGGGCATGATGGAAT |
|  | rs1532268 | ACGTTGGATGAGGAGATAAGTGGCGCACTC | ACGTTGGATGTGTGTAGCAGCTCTGACTTC | GGCATCACCTGCATCCT |
|  | rs2287780 | ACGTTGGATGAGCTGTGCAGTAAACAAGGG | ACGTTGGATGAGGAGGAGATCCAACAAGCA | CAAGGGGCAGCCGATTATAGC |
|  | rs9332 | ACGTTGGATGCCCTCTGTAGGCAATTATCC | ACGTTGGATGGGTTATTTGTTACTAAAGC | GGCAATTATCCTAAAATATTTTTTAT |
|  | rs8659 | ACGTTGGATGGCAAATGTCCCAAAATTCTG | ACGTTGGATGCACTCTGGCATATGATTTATC | CAAAATTCTGAAATTGTGACTT |
|  | rs10520873 | ACGTTGGATGAGGCTTTTGATCCTTTTGAG | ACGTTGGATGCACAGCCACAAAAACTTACC | ATAAAGATCTGAAAGAAATGGCATAA |
| ***SHMT1*** | rs643333 | ACGTTGGATGGTAGTGCAGCTGCGCACCT | ACGTTGGATGAATCTTGGTCCACACACCAG | TGGCAGGGACCTGCAGAACTGACCC |
| ***TYMS*** | rs9967368 | ACGTTGGATGACCCCTTATCTTAACCCAG | ACGTTGGATGTGGGTGACAGAGCCGTATG | ACCCAGATATTCCTTTCTATT |
|  | rs2853741 | ACGTTGGATGGGAAACAGATCTCAAACAGC | ACGTTGGATGTTCTCTAAGCCAGCAGCACA | CACAGATCTCAAACAGCAGTTTTGT |
|  | rs699517 | ACGTTGGATGACTTTTACCTCGGCATCCAG | ACGTTGGATGTGGCTGTTTAGGGTGCTTTC | CTAAAGACTGACAATATCCTTC |
