# Supplementary material for: One-Carbon Metabolism Pathway Gene Variants and Risk of Clear Cell Renal Cell Carcinoma in a Chinese Population
Source: PLoS One. 2013 Nov 21;8(11):e81129. doi: 10.1371/journal.pone.0081129 (PMC3837692; doi:10.1371/journal.pone.0081129)
Supplement: Table S2 — Polymorphism in one-carbon metabolism genes and clear cell renal cell carcinoma. (DOC). (DOC) [file pone.0081129.s002.doc]

**Table S2.** Polymorphism in one-carbon metabolism genes and clear cell renal cell carcinoma

|  | Position | MAF | Cases, n (%) | Controls, n (%) | *P*-value^a^ | Adjusted OR (95% CI)^b^ | *P* trend^a^ | HWE |
| --- | --- | --- | --- | --- | --- | --- | --- | --- |
| *MTHFR* |  |  |  |  |  |  |  |  |
| rs3737965 | promoter | 0.1 |  |  |  |  |  |  |
| CC |  |  | 728(84.8) | 854(85.0) | 0.990 | Ref. | 0.886 | 0.257 |
| CT |  |  | 123(14.3) | 142(14.1) |  | 1.09(0.83-1.42) |  |  |
| TT |  |  | 8(0.9) | 9(0.9) |  | 0.98(0.36-2.67) |  |  |
| TT vs. CC+CT |  |  |  |  | 0.892 | 0.97(0.36-2.62) |  |  |
| CT+TT vs. CC |  |  |  |  | 0.935 | 1.08(0.83-1.40) |  |  |
| rs4846049 | 3’UTR | 0.2 |  |  |  |  |  |  |
| GG |  |  | 611(71.1) | 726(72.2) | 0.867 | Ref. | 0.601 | 0.086 |
| TG |  |  | 220(25.6) | 248(24.7) |  | 1.05(0.85-1.31) |  |  |
| TT |  |  | 28(3.3) | 31(3.1) |  | 1.06(0.62-1.82) |  |  |
| TT vs. GG+TG |  |  |  |  | 0.830 | 1.03(0.61-1.77) |  |  |
| TG+TT vs. GG |  |  |  |  | 0.596 | 1.05(0.85-1.30) |  |  |
| rs4846048 | 3’UTR | 0.102 |  |  |  |  |  |  |
| AA |  |  | 702(81.7) | 817(81.3) | 0.801 | Ref. | 0.920 | 0.376 |
| AG |  |  | 149(17.4) | 181(18.0) |  | 0.93(0.73-1.19) |  |  |
| GG |  |  | 8(0.9) | 7(0.7) |  | 1.43(0.50-4.05) |  |  |
| GG vs. AA+AG |  |  |  |  | 0.572 | 1.44(0.51-4.06) |  |  |
| AG+GG vs. AA |  |  |  |  | 0.812 | 0.95(0.75-1.21) |  |  |
| *MTR* |  |  |  |  |  |  |  |  |
| rs1050993 | 3’UTR | 0.222 |  |  |  |  |  |  |
| GG |  |  | 588(68.5) | 660(65.7) | 0.270 | Ref. | 0.374 | 0.387 |
| GA |  |  | 240(27.9) | 314(31.2) |  | 0.86(0.70-1.06) |  |  |
| AA |  |  | 31(3.6) | 31(3.1) |  | 1.08(0.63-1.83) |  |  |
| GG vs. AA+AG |  |  |  |  | 0.529 | 1.12(0.66-1.89) |  |  |
| AG+GG vs. AA |  |  |  |  | 0.203 | 0.88(0.72-1.08) |  |  |
| rs6676866 | 3’UTR | 0.467 |  |  |  |  |  |  |
| GG |  |  | 253(29.5) | 300(29.9) | 0.332 | Ref. | 0.340 | 0.163 |
| GT |  |  | 423(49.2) | 518(51.5) |  | 1.01(0.81-1.25) |  |  |
| TT |  |  | 183(21.3) | 187(18.6) |  | 1.21(0.92-1.59) |  |  |
| TT vs. GG+GT |  |  |  |  | 0.146 | 1.20(0.95-1.52) |  |  |
| TT+GT vs. GG |  |  |  |  | 0.851 | 1.06(0.86-1.30) |  |  |
| rs2282368 | 3’UTR | 0.227 |  |  |  |  |  |  |
| CC |  |  | 476(55.4) | 553(55.0) | 0.236 | Ref. | 0.618 | 0.387 |
| CT |  |  | 316(36.8) | 392(39.0) |  | 0.94(0.77-1.14) |  |  |
| TT |  |  | 67(7.8) | 60(6.0) |  | 1.28(0.87-1.87) |  |  |
| TT vs. CC+CT |  |  |  |  | 0.118 | 1.32(0.91-1.91) |  |  |
| CT+TT vs. CC |  |  |  |  | 0.867 | 0.98(0.81-1.19) |  |  |
| *MTRR* |  |  |  |  |  |  |  |  |
| rs1532268 | missense | 0.122 |  |  |  |  |  |  |
| GG |  |  | 653(76.0) | 745(74.1) | 0.613 | Ref. | 0.411 | 0.580 |
| GA |  |  | 191(22.2) | 243(24.2) |  | 0.89(0.71-1.12) |  |  |
| AA |  |  | 15(1.8) | 17(1.7) |  | 0.95(0.46-1.94) |  |  |
| AA vs. GG+GA |  |  |  |  | 0.928 | 0.98(0.48-2.00) |  |  |
| GA+GG vs. AA |  |  |  |  | 0.348 | 0.90(0.72-1.12) |  |  |
| rs2287780 | missense | 0.122 |  |  |  |  |  |  |
| CC |  |  | 608(70.8) | 723(71.9) | 0.667 | Ref. | 0.468 | 0.276 |
| CT |  |  | 231(26.9) | 264(26.3) |  | 1.08(0.87-1.33) |  |  |
| TT |  |  | 20(2.3) | 18(1.8) |  | 1.28(0.66-2.47) |  |  |
| TT vs. CC+CT |  |  |  |  | 0.413 | 1.26(0.65-2.44) |  |  |
| CT+TT vs. CC |  |  |  |  | 0.581 | 1.09(0.88-1.34) |  |  |
| rs8659 | 3’UTR | 0.481 |  |  |  |  |  |  |
| TT |  |  | 208(24.2) | 243(24.2) | 0.617 | Ref. | 0.481 | 0.216 |
| TA |  |  | 460(53.6) | 520(51.7) |  | 1.02(0.68-1.21) |  |  |
| AA |  |  | 191(22.2) | 242(24.1) |  | 0.92(0.61-1.18) |  |  |
| AA vs.TT+TA |  |  |  |  | 0.347 | 0.91(0.62-1.09) |  |  |
| TA+TT vs.TT |  |  |  |  | 0.986 | 1.00(0.79-1.35) |  |  |
| *SHMT1* |  |  |  |  |  |  |  |  |
| rs643333 | promoter | 0.083 |  |  |  |  |  |  |
| CC |  |  | 749(87.2) | 879(86.6) | 0.782 | Ref. | 0.627 | 0.693 |
| CA |  |  | 108(12.6) | 131(13.0) |  | 0.95(0.72-1.27) |  |  |
| AA |  |  | 2(0.2) | 4(0.4) |  | 0.63(0.11-3.46) |  |  |
| AA vs. CC+CA |  |  |  |  | 0.530 | 0.63(0.11-3.49) |  |  |
| CA+AA vs. CC |  |  |  |  | 0.690 | 0.94(0.71-1.25) |  |  |
| *TYMS* |  |  |  |  |  |  |  |  |
| rs9967368 | promoter | 0.478 |  |  |  |  |  |  |
| CC |  |  | 292(34.0) | 354(35.2) | 0.852 | Ref. | 0.596 | 0.996 |
| CG |  |  | 421(49.0) | 485(48.3) |  | 1.14(0.92-1.40) |  |  |
| GG |  |  | 146(17.0) | 166(16.5) |  | 1.09(0.82-1.44) |  |  |
| GG vs. CC+CG |  |  |  |  | 0.782 | 1.02(0.80-1.32) |  |  |
| CG+GG vs. CC |  |  |  |  | 0.578 | 1.21(0.92-1.37) |  |  |

^a^ Two-sided χ2-test for either genotype distributions or trend between the cases and controls.

^b^ genotype-specific ORs were adjusted for age, gender, BMI, smoking status, drinking status, diabetes and hypertension in logistic regression model.
